# The Threat of COVID-19 and Job Insecurity Impact on Depression and Anxiety: An Empirical Study in the USA

**DOI:** 10.3389/fpsyg.2021.648572

**Published:** 2021-08-13

**Authors:** Bojan Obrenovic, Jianguo Du, Danijela Godinic, Mohammed Majdy M Baslom, Diana Tsoy

**Affiliations:** ^1^School of Management, Jiangsu University, Zhenjiang, China; ^2^Faculty of Philosophy, University of Zagreb, Zagreb, Croatia; ^3^Hunan University, Changsha, China; ^4^School of Media and Communication, Shanghai Jiao Tong University, Shanghai, China

**Keywords:** COVID-19, depression, anxiety, job insecurity, EPPM threat, EPPM model, mood disorders

## Abstract

As the coronavirus disease 2019 pandemic causes a general concern regarding the overall mental health of employees worldwide, policymakers across nations are taking precautions for curtailing and scaling down dispersion of the coronavirus. In this study, we conceptualized a framework capturing recurring troublesome elements of mental states such as depression and general anxiety, assessing them by applying standard clinical inventory. The study explores the extent to which danger control and fear control under the Extended Parallel Processing Model (EPPM) threat impact job insecurity, with uncertainty phenomenon causing afflicting effect on the experiential nature of depression heightened by anxiety. With the aim to explore the job insecurity relationship with anxiety and depression, and measure the impact of EPPM threat, an empirical study was conducted in the United States on a sample of 347 white collar employees. Demographic data, EPPM threat, job insecurity, anxiety, and depression data were collected *via* a standardized questionnaire during the coronavirus disease 2019 (COVID-19) pandemic. The questionnaire consisting of multi-item scales was distributed online. All the scale items were evaluated on a 5-point Likert scale. SEM software AMOS version 23 was used to perform confirmatory factor analysis with maximum likelihood estimation. In the structural model, relationships between the threat of COVID-19, job insecurity, anxiety, and depression were assessed. The findings of the study suggest that job insecurity has a significant impact on depression and anxiety, whereas the threat of COVID-19 has a significant impact on depression. Mediating effects of job insecurity and EPPM threat impact on anxiety were not established in the study. The study contributes to the apprehension of the repercussions of major environmental disruptions on normal human functioning, and it investigates the effects of self-reported protective behaviors on risk perception. The study also explains the underlying mechanisms of coping behavior as possible antecedents to mental disorders. When subjected to stressful events, heightened psychological arousal causes physical and psychological challenges of affected employees to manifest as behavioral issues.

## Introduction

The coronavirus disease 2019 outbreak caused a general concern regarding overall mental health implications on employees worldwide. The coronavirus disease 2019 (COVID-19) pandemic hastened the most abrupt and profound economic contraction the United States has experienced since the Great Depression, leaving millions of Americans jobless and destitute, counting the rise of the unemployment rate and operating below the full capacity for a prolonged period (Parolin et al., 2020). As temporary lockdowns, mass layoffs, and economic crisis became an objective threat to public welfare, psychologists and psychiatrist around the world warned about severe long-term consequences such conditions will have on psychological well-being and increase in cases of mental disorders (Stein et al., 2013; Godinic et al., 2020). Autonomy and a sense of control over the environment and one's overall life situation are essential components of psychological well-being, meaning unpredictable and uncontrollable nature of the current pandemic is bound to have a detrimental effect on mental health (Huber et al., 2011; Lo and Cheng, 2014). Mass protests against economic closing and extreme social distancing measures resulted in bitterness, intentional breaching of protective policies, and willingness to risk contagion, and finally, an increase in dissatisfaction, resentment, and public despair. These actions stress the relevance of preservation or restoration of the very resources employees find crucial for their healthy functioning (Brashers, 2001). According to existing studies, the need for socializing and companionship can predict stress levels during a crisis (Casale and Flett, 2020). The damages to the human psyche have manifested in a drastic increase in general anxiety, insomnia, depression (Giorgi et al., 2020), and suicidal ideation.

In this study, we conceptualized a framework capturing recurring troublesome elements of states such as depression and general anxiety, assessing them by applying standard clinical inventory, as we found the self-assessment and self-appraisal of the ephemeral plight sheds light on the alternations and breakdown in experiences shadowed by the abrupt danger of COVID-19 (Shirahmadi et al., 2020). As Clarke (2002) posits, according to common belief regarding human nature, in peril, people act alarmed when in crowds. These make them subjective to reckless conduct in search of self-preservation, possibly endangering the survival of the rest. The observations of bearings of the prevailing fear caused by general uncertainty, especially within the limits of economic context, allowed hypothesizing a link between the central concept of job security and its effect on psychological impairment, namely, depression and anxiety. These were found to be the root of social and, consequently, individual self-disintegration and cause of emotional, psychological, and occupational disengagement designated by the loss of interest, self-doubt, and negative appraisals of one's overall life situation. We posit that experiential alternations, as reported by the respondents, ranging from suffering, low-mood, disinterest, impairment, and disability in pursuing one's goals, professional or otherwise, as well as an inability to find previous activities meaningful are the result of maladaptive responses to the COVID-19 cautionary campaigns. As such, the study sets a dual objective, i.e., of exploring the extent to which danger control and fear control under the EPPM threat impact job insecurity, with uncertainty phenomenon causing afflicting effect on experiential nature of “going through” depression heightened by anxiety, and broadening the emergent literature on crisis management for sustaining psychological welfare as required by the immediate threat of COVID-19 pandemics. Van Bavel et al. (2020) noted that the research topic selection centring on underlying mechanisms of behavioral response to pandemics is exceptionally relevant for informing the authority, governments, epidemiologists, social scientists, and national headquarters on how threat, anxiety, perceived danger, and social influences guide the behavior.

In some respect, the healthcare system has been subjected to analogous epidemiological hazards and has gone through similar case scenarios with Avian flu, SARS, and MERS outbreaks. We also draw from lessons on potential psychological adversity induced by other societal and economic global disasters, such as the Great Depression. The coronavirus is unique, as it encompasses the cumulative disruption of all well-being-related aspects—social, physiological, psychological, and monetary. As the emergent situation is relatively new, there is a clear lack of indication on how to proceed best with prospective workplace prevention and protection (Zhao and Wu, 2021). This study provides a preliminary exploration of potential key antecedents of mental resilience of employees and risk factors for the occurrence of mental disorders under prolonged stress exposure. While most studies on risk communication are focused on the influence of media on employees response formation (Chong and Choy, 2018; Garfin et al., 2020; He et al., 2020; Manzoor and Safdar, 2020; Tsoy et al., 2021), we have undertaken to investigate how threat perception and perceived efficacy under the ongoing exposure to job uncertainty can precipitate mood disorders. There is lack of support in the literature that would explain how exposure to hazard-related information influences the formation of adaptive or maladaptive coping strategies of employees aimed at psychological well-being retention. Also, although many studies have previously corroborated that uncertainty influences psychosocial and occupational functioning (Aguiar-Quintana et al., 2021; Blanuša et al., 2021; Ganson et al., 2021; Ruffolo et al., 2021), there are not many studies in the field of occupational psychology examining how job uncertainty leads to protective motivation when EPPM perpetuates the insecurity.

The findings will be of great use for aligning the scientific and epidemiologic communication to existing public concerns, comprehending how external stimuli influence internal states of individuals, steering the public toward ethical decision-making and healthy coping strategies (Lasbeur et al., 2020).

## Theory And Research Model Development

### The Threat of COVID-19 (EPPM Threat)

We gain much by understanding how rational considerations, i.e., efficacy beliefs and emotional reactions such as fear, inform and determine behavioral decisions. To be consequential and influential, educational propagations regarding pressing health matters that may not be entirely eradicated but whose adverse effects can be significantly reduced should be born out of episteme focusing on extensive behavioral change. The EPPM, also known as Threat Management or Fear Management, accounts for particular modifications in active engagement of individuals in responding to significant health-threatening events. Etymologically, EPPM tenets were borrowed from three preceding models, namely, the fears-acquired drive model (Hovland et al., 1953), parallel process model (Leventhal, 1970), and protection motivation theory (PMT; Rogers, 1975, Maddux and Rogers, 1983). The Hovland et al. (1953) model accounts for both adaptive and maladaptive responses. The leading idea stems from a theory that human behavior can be interpreted through learned responses and rewards, much in line with behaviorist reasoning. Individuals learned to fear a threat as an incentive to reduce the unpleasant state of fear by assuming a particular action.

Effective actions provided a habitual response for all future analogies. The fear intensity determined whether the adaptive action with “reassuring recommendations” or “maladaptive” defensive avoidance will be assumed. According to the parallel process model, the duality of responses implied the existence of two autonomous reactions to fear—a cognitive danger control process devising a strategy to avert the threat, and an emotional fear control process rooted in retraction and avoidance. Finally, protection motivation theory (Maddux and Rogers, 1983) singled out four components of a threat message. The first referred to the probability of a threat, the second concerned with severity, the third was related to response effectiveness, and the fourth referred to self-efficacy. Optimal protection response was given when all the four cognitions were highly intense and, thus, provided increased protection motivation, resulting in adaptive behavioral change (Popova, 2012). Such application is ideal in the advent of COVID-19, as many campaigns on prevention and reduction emerge daily, accompanied by specific national, local, and organizational policy measures and recommendations (Lasbeur et al., 2020; Raude et al., 2020). The EPPM provides an action-oriented framework for emotion acting as a behavioral predictor rather than a mere mental state devoid of action (Shirahmadi et al., 2020). The rationale for EPPM is that threat intensity influences the decision to act, and that confidence in the viability of effective prevention of the threat determines the action. The two mental processes, namely, danger control and fear control, lead to adaptive and maladaptive responses. The successful exploration hinges on the imperative that a severe threat or a hazard of COVID-19 is taken seriously by the target recipients, to whom the appeal is presented. The EPPM relies on a persuasive exposure to messages relating to fear and the subsequent introduction of an adequate response that aids to avert the threat (Kotowski et al., 2011). Combining the threat presentation and the response action is expected to stimulate protective motivation. Such communication mechanism is often used by healthcare adversaries in a range of campaigns, such as health advocating (Dutta-Bergman, 2005), early breast cancer detection (Kaplowitz et al., 2002), alcohol warnings (Campo et al., 2005), caution on tobacco usage repercussions (Sanderson and Wardle, 2005), and chronic disease awareness (McKay et al., 2004).

Furthermore, the EPPM predicts that when a threat is considered irrelevant, the subject will lack the incentive to accept the message and fail to enact the action. Perceived self-efficacy in the current context involves the perception of individuals of their capability to enact the defensive response, i.e., to practice social distancing, avoid gatherings, wear protective masks, and frequently wash hands using hand sanitizers. Response efficacy then refers to the perception of individuals that said instructions would reduce and prevent the virus spread. The successful campaign should balance the threat perception and response efficacy, i.e., high levels indicate that while danger is acknowledged, it is perceived as being conveniently and simply avoided.

Most common strategic measures to contain coronavirus spread propose practicing social distancing, wearing protective masks, avoiding large gatherings, frequently washing hands, using hand sanitizer, and practicing e-education and remote working. It is only logical that the fear posed by novel virus threat is excessive. With part of the public still doubting the severity of the disease, protesting about novel policies and confinement, as well as commuting and mobility restrictions, policymakers are coming up with caution techniques and warning campaigns containing educational materials, showcasing photos of hospitalized individuals and devastated family members, exposing long-term consequences.

Such maneuver aims to educate by increasing public awareness and amplifying the threat while providing advice and recommendations on avoiding the infection. The communication of repercussions concerns health side effects and informs the public about the danger national economies and local businesses face, with a realistic possibility of prevalent unemployment. One of the most controversial measures to save the global economy and avoid further closures and layoffs was implementing remote working.

Both depression and anxiety were related to impaired psychosocial and occupational functioning (Alonso et al., 2004; Druss et al., 2009; Hussain et al., 2011). With the ambiguity surrounding roles and responsibilities of employees, as tasks and priorities are rapidly changing in pace with the alternations in policies and company activities in response to progression in pandemics, contention and disagreements are bound to arise. Much of the protest to changing operational approach according to volatile market appears as a fallout caused by unfamiliarity and hostility of emerging threat, as well as affliction of the continuous fear and instability surrounding current employment of employees, future employability, loss of income and social support, work-related monetary and non-monetary benefits, as the threat may result in the deprivation of vital psychological resources (Jofre-Bonet et al., 2018). Following the EPPM, we investigated whether the threat of COVID-19-induced fear impacts job insecurity. Therefore, we theorize:

Hypothesis 1: The threat of COVID-19 (EPPM threat) leads to job insecurity.

### The Relationship Between Uncertainty and Depression

Previous qualitative studies concentrated on interviewing depressed individuals have found that the respondents felt “unpredictability and lack of control over something that has a life of it's own…” (Karp, 1996, pp. 124–5). Interestingly enough, the very absence of control and autonomy that characterizes depressive state during the depressive episode is, at the same time, considered to be the trigger for the mood disorder. Causal relationship should, therefore, be evident, as the same feature is the cause as well as prevailing quality of said condition. Furthermore, the notion that depression influences the Self is not new, as psychoanalytic account has long ago identified depressive swing, or melancholy as Freud named it, as an acute feeling of suffered loss that eventually leads to grief and self-hatred (Freud, 1957). Moreover, agency of the Self is embodied in human experience as world-dependent, as philosophers, namely, Freud (1957), Heidegger (1927/1962), and Fuchs (2002), long supposed, the contextual societal or economic life situation and the environmental causation of psychological disorder cannot be denied. More specifically, changes brought about by disruptions in regular social, political, and economic functioning are bound to affect psychological well-being (Reichert and Tauchmann, 2011). The more negative dimensions of said changes are pronounced, the more paralyzing and detrimental they are for the well-being of individuals. Several studies found that financial stressors related to economic crises and perceived, prospect, or actual job loss precipitate a strain on mental health (Catalano et al., 2011; Althouse et al., 2014; Jesus et al., 2016), causing depression, anxiety, and suicidal ideation. Such conclusions were mostly derived from the aftermath of the 2008 financial crisis (Green, 2011; Huber et al., 2011; Flint et al., 2013), and following the Great Recession and the increase in the recorded instances of mental disorders (Lo and Cheng, 2014; Phillips and Nugent, 2014).

According to DSM, the concept of disorder encompasses a clinically significant behavioral or psychological syndrome or pattern occurring in an individual that is associated with present distress (e.g., a painful symptom) or disability (i.e., impairment in one or more critical areas of functioning) or with a significantly increased risk of suffering death, pain, disability, or a significant loss of freedom (American Psychiatric Association, 2000, xxxi). Nevertheless, we intentionally divulge from such categorical classification of depression, especially considering the underlying notion that depression is biologically embedded in specific brain dysfunction. Such rigid account ignores the circumstantial sources and explanandum of depressive episodes, some of which, when considered on a societal level, even bears the title of “The major depression” as a historical reference. Many previous studies generated substantial empirical evidence associating economic collapse, catastrophes, natural disasters, epidemics (Liao et al., 2014) and the like with increase in the recorded number of instances of depression, anxiety, paranoia, chronic exhaustion, substance abuse, and suicidal ideation (Almeida and Xavier, 2013; De Vogli et al., 2013; Branas et al., 2015).

Such characterization indicates that external environmental factors, such as distress, risk of suffering a loss, or being depleted of freedom, are the root cause of psychological impairment. Apart from autonomy and sense of control over one's environments, other psychological well-being constituents were previously identified as the need for relatedness, belonging, support, accomplishment, self-confidence, achievement, professional and personal growth, and individual advancement. These stem from safe, secure, fulfilling, purposeful, and meaningful employment, where occasional disturbances or changes do not affect the overall satisfaction of employees.

### Job Insecurity

Insecurity- or uncertainty-defining features comprise ambiguous, complicated, volatile, inconsistent, and unpredictable conditions, prompting an individual and the general public to dwell on their competency and ability to reason, and reliability of the knowledge and information available (Brashers, 2001). We draw from the conservation of resources theory to account for the adverse effects of uncertainty on psychological health, prompting psychological distress, arousal, fear, and exhaustion, thus causing the increase in depression and anxiety instances. According to COR, every insecurity is consequential for the deprivation of resources, as one invests an extensive amount of energy in devising coping mechanisms to decrease stress intensity and sustain the impression of stability (Vander Elst et al., 2014). Previous studies have found coping behavior to mediate the relationship between economic stress variables and psychological health variables (Stein et al., 2013). Job security is characterized by Herzberg (1968) as a state wherein the organization provides a stable environment and a guarantee of employment, including all the corresponding benefits, such as seniority rights, retirement security, steady income, and an opportunity for self-development and self-actualization. Job security is commonly characterized as persistent certainty regarding one's employment situation, involving financial, social, and economic stability through continued employment within the organization or a particular profession (Herzberg, 2017).

Furthermore, relatedness and the desire to stay connected are especially relevant during catastrophic events and crises, as pressing circumstances amplify the need for social support (Ryan and Deci, 2000). Burke (2020) suggested identity as an umbrella term regarding what it means to be who one is in a role he/she assumes, therefore referring to role identities, social identities, and personal identities; all of which are, consequently, threatened by uncertain and turbulent situations. One way these can be threatened by job insecurity is through the very notion of social identity, as revealed in the sense by individuals of pertaining to a broader community, thus making the concept dependent on societal conditions. Job uncertainty brings to the surface fear of poverty, leading to marginalization, stigmatization, and social exclusion (Rafi et al., 2019). It is relatively easy to recognize the association between sustaining continuous employment and fulfillment of basic human needs. Individuals strive for self-actualization, whereby they are provided with the opportunity to achieve their full potential and engage in creative endeavors, further developing their creative skills. Self-esteem needs refer to one's social status and recognition, ensuring the desired social position related to accomplishment and prestige. The need for affiliation encompasses one's intimate relationships and concerns a social network comprised of friends, family, partners, coworkers, subordinates, team members, supervisors, and mentors, including accompanying support, counseling, therapy, and guidance. Isolation, recess in social interaction and social exclusion, perceived as losing social control and valuable resources, causes severe damage to the psyche. Safety needs directly concern job security, as job is the central component of psychological well-being from which all other resources derive. Apart from providing security regarding continuous employment, future career prospects, financial income, and non-monetary benefits, it also serves as a tool to satisfy essential physiological needs for survival, providing access to food, water, and housing.

In contrast, continuous adverse, volatile, and unpredictable disruptions affecting the work environment pose a threat for distress, psychological strain, extreme fear, panic event, ambiguity, loss of interest, disengagement, falling behind on performing regular work tasks, exhaustion following extensive worrying, disturbed interaction, loss of faith in leadership and organizational sustainability, and therefore, one's existential security. Job security is a means for securing the most essential resources (Thompson et al., 2017; Huang et al., 2020). Consequently, we conclude the following hypothesis:

Hypothesis 2: Job insecurity leads to depression.

### Anxiety

The phenomenon of anxiety as the uncovering of the ultimate meaning of life and death, as well as the existential dread or the existential stratum, does not exclusively fall under the domain of psychology and psychiatry but has, long before the establishment of said disciplines, inhabited the minds of some of the greatest philosophers (Kierkegaard, 1843/1954, 1849/1954; Sartre, 1939/1994, 1989; Husserl, 1954). For example, Tillich (1952a,b) distinguished between fear and anxiety, maintaining that fear has a definite object while anxiety does not. Moreover, existential anxiety occupying the central place in the theoretical and philosophical discussions was closely associated with fear, where the fear of death was interpreted as the reification of anxiety. Therefore, when contemplating fate, death, and contingency of life, the Self is preoccupied with its fate.

Theoretical assumptions were further supported by empirical research, including survey studies and detailed interviews that sprouted out of the terror management theory (TMT). TMT assumes that all anxiety eventually springs out of fear of absolute annihilation, proceeding from a drive for self-preservation combined with an awareness of mortality (Solomon et al., 1991). When a safeguard to this awareness cannot be maintained, psychopathology arises. According to the empirical study of Cypryańska and Nezlek (2020) on emotional reactions, coping behaviors, and willingness to undergo economic sacrifices concerning COVID-19, the level of anxiety, hopelessness, and panic was examined. They found that threat to the Self was predictive of emotions and coping, while anxiety was the most reliable determinant of spread prevention and economic sacrifice, while panic predicted self-preservation. Furthermore, Stanton et al. (2020) found a correlation between anxiety and stress during COVID-19 with sleep and substance abuse.

In psychiatric terms, following the DSM, the symptoms of depression result in clinically significant distress and impairment in social, occupational, or other important life domains. Agitation following depression may be caused by underlying anxiety; therefore, diagnostic tools regularly include anxiousness to establish the presence and severity of depression (Svenaeus, 2007, 2008). Phenomenologically taken, the diagnostic scheme of depression consists of an acute alternation of embodiment and pathologically estranged alienation from previous activities, professional or otherwise, social, educational, family, and marital. Building on the (1962, 1971) motivational hierarchy of human needs by Maslow, the crisis prompted by the COVID-19 pandemic results in physiological, relational, esteem and self- determination, and most importantly, safety needs not being met, being restricted or cutoff because of changes in external circumstances and socio-economic changes, such as economic collapse following the global pandemic. Limited access to a job, finances, and healthcare benefits causes severe psychological impairment, anxiety, and corresponding depression (Fabian, 2013; Flores et al., 2017; Blustein et al., 2019). Such states only increase because of the perpetuating absence of clinical treatment, further damaging well-being (Lai et al., 2020).

Hypothesis 3: Job insecurity leads to anxiety.

Furthermore, social isolation, prevention of gatherings, closing of restaurants and coffee shops, and other entertainment facilities, introducing police hours and restricting commuting depleted many individuals of much needed social support (Jahangiry et al., 2020). As mentioned before, one of the fundamental human needs for affiliation suffered a severe blow, and this left a psychological strain on people (Killgore et al., 2020a,b; Tull et al., 2020). Social identities were shaken, and group affiliations broke, leaving people dealing solely with dark chronicles and depressing thoughts. In adopting a defensive response, people will base the judgment according to information gathered through official sources and personal social network channels (Zhao and Wu, 2021). Appraisals and facts concerning threat severity and available resource to form an effective coping system will function either in support of or against the implementation of mental health interventions, as they can be perceived as valuable or ineffectual (Pollock et al., 2020). An adaptive response to campaigns launched would consist of restraining oneself from breaking the rules and socializing only when necessary, otherwise using the online channels for working, networking, and interacting. Furthermore, it would include the apprehension that such measures, including even lockdowns, benefit the entire community.

Regarding guidance of the EPPM health campaign, individuals would ward off incoming depression by utilizing widely available services for psychological support and free counseling sessions and therapeutic advice, available *via* phone or online. The simplicity of the response depends on individual self-efficacy belief (Van Bavel et al., 2020). Enacting the response seems simple enough, yet among dependent personalities, psychologically imbalanced and prone to mood disorders individuals, it may easily trigger depression. Deprived of reactions from other people and lacking validation from a previously established intersubjective network, unstable personalities may even feel as they are seizing to exist. A maladaptive response would consist of excessive resistance or avoidance that would finally result in alienation and loss of interest for any previously meaningful activity (Aguiar-Quintana et al., 2021; Ganson et al., 2021). Life itself may even become meaningless when the fear overweighs the efficacy belief. Therefore, we hypothesize the following:

Hypothesis 4: The threat of COVID-19 (EPPM threat) leads to depression.

Impulsion to constantly check news outlets stressing the hazards of the virus, counting the number of infected, unemployed, businesses closed, and highlighting the financial debt leaves one not only fearing the virus but rather dreading its extensive consequences even long after the pandemic is contained. In the United States, GDP plunged in the second quarter of 2020, leaving millions of Americans unemployed and permanently laid off. The preventive measure Coronavirus Aid, Relief, and Economic Security Act (CARES) amounting to $2.2 trillion was launched in March; yet, according to a study on poverty and social policy conducted by the researchers of Columbia University, the number of poor people has grown by eight million since May (Parolin et al., 2020). Many sudden and uncontrollable disasters induce extreme anxiety, causing fear, anxiousness, irritability, aggression, suicidal ideation, substance abuse, and even psychosis. Previous studies have identified many individuals to have developed a full-blown general anxiety disorder or PTSD in the aftermath of a sudden crisis. While the educational purpose is to raise awareness and provide guidelines for safe navigation through the new normal, the fear provoked by absorbing projected images and news communicated may become too intense, causing individuals to develop maladaptive coping mechanisms (Witte and Allen, 2000), such as excessive avoidance that gradually turns into an avoidance personality disorder. One may develop obsessive-compulsive disorder, always under attack of intrusive thoughts and harmful actions such as, for instance, excessive hand-washing and scrubbing resulting in physical harm. Alternatively, the howling fear and anticipation may become so exhaustive that they paralyze the individual, rendering one temporarily incapacitated for work, thus increasing one's chance to lose a job. Excessive fear of threat makes the threat seem imminent (Cole et al., 2013; Zhao and Wu, 2021). The foundation of a healthy economy is a healthy workforce, yet stressing over the recession, budgetary cutback, salary reduction, and making drastic economic sacrifices to combat the virus induces profound anxiety. The danger control, e.g., the adaptive response, would comprise strictly complying with the instructions of the epidemiologists provided by EPPM propagation, as it is abundant with useful advice on how to apply lifestyle changes to thwart the infection successfully. Moreover, by relying on national aid programs, packages and relief, anxiety regarding one's access to job and essential life supplies would expectedly decrease (Carmassi et al., 2020). From this, the following is concluded:

Hypothesis 5. The threat of COVID-19 (EPPM threat) leads to anxiety.

## Materials and Methods

### Participants and Procedure

The purposive sampling technique was employed in the study, which conducted an online cross-sectional survey distributed among employees in companies and institutions located in the United States. Respondents were working in information technology, electronics, medicine, and biochemistry field. The survey focused on white collar workers to increase the homogeneity of the research sample. All participants were informed of the purpose and anonymity of the investigation and their right to terminate participation at any time when needed. Data collection was conducted from May to August 2020, during COVID-19 pandemic. Demographic data, EPPM threat, job insecurity, anxiety, and depression data were collected *via* a standardized questionnaire. The questionnaire consisting of multi-item scales was distributed online to collect data. All the scale items were evaluated on a 5-point Likert scale. A total of 500 emails were sent to employees *via* the company HR department. A total of 413 respondents accessed the survey link and completed the questionnaire. All the incomplete replies were eliminated from the data set. Only the completed questionnaires were used in the analysis process. A final sample prepared for analysis consisted of 347 employees.

After eliminating the missing values, the analysis was performed on the sample of 347 respondents, of which 51.5% were males and 48.5% were females. Most of the respondents were over 50 years old, with 70.8, and 25.6% were between 30 and 50 years old, respectively. Only 3.6% of the respondents were under 20 years old. In terms of educational background, over 90% of the participants have a bachelor degree or above. Specifically, 5.2% of the respondents have a bachelor degree, 85.3% hold a postgraduate and PhD degree. The remaining 8.5% hold a high school degree. As for employment status, among all the respondents, 64.8% worked 40 h or more per week, and the others worked <40 h per week.

### Measurements

#### Extended Parallel Process Model Threat

An extended parallel process model (EPPM) was utilized to explore the impacts of COVID-19 on individuals. There were two mental processes in this model, danger control and fear control. The process of danger control leads audiences to respond adaptively, such as accepting a message, while the fear control process caused them to make maladaptive responses, such as rejecting the message (Witte, 1992). The scale consists of six items that measure the perceived severity and susceptibility of COVID-19 on people.

Sample items include: “I am at risk for COVID-19,” “I believe that COVID-19 is a serious threat to my health.” The response options ranged from “strongly disagree” to “strongly agree” on a 5-point Likert scale. The internal inconsistency was at an acceptable level, measured with Cronbach's alpha (α = 0.78).

### Job Insecurity

The job insecurity (JIS) scale was adopted from De Witte (2000). This instrument measured the degree of JIS from two directions: one was the impact of external environment of an individual on his or her job insecurity level, and the other combined both cognitive appraisal and affective appraisal, that is, the impact of the personal perception of these situations on his or her job insecurity level. Four items assessed JIS, and as follows: “(1) Chances are, I will soon lose my job, (2) I am sure I can keep my job, (3) I feel insecure about the future of my job, and (4) I think I might lose my job in the near future.” The scale showed an acceptable level of reliability (α = 0.82). The response options ranged from “strongly disagree” to “strongly agree” on a 5-point Likert scale.

### Anxiety

The seven-item Generalized Anxiety Disorders (GAD-7) scale is commonly used to measure general worry and anxiety symptoms in different settings and populations. The GAD-7 scale has shown adequate reliability in past studies (Tong et al., 2016; Wang et al., 2018), and the scale reliability was confirmed in this study (α = 0.91). The GAD-7 scale consists of seven items, and each item was scored on a 5-point Likert scale ranging from “1” represented “not at all” to “5” represented “nearly every day.” In addition, the respondents were asked to evaluate how often they have been bothered by the specific problems over the last month. Sample items include: “Not being able to stop or control worrying” and “Becoming easily annoyed or irritable.”

### Depression

The Center for Epidemiology Scale for Depression (CES-D) (Radloff, 1977), developed by the National Institute of Mental Health for epidemiological research, was used to measure the frequency of depression symptoms. The scale consists of 20 items and five possible answers in a Likert format for respondents, from “1” for “rarely or none of the time (<1 week)” to “5” for “almost or all of the time (the whole month).” The CES-D scale consists of four independent factors: depressive affect, somatic symptoms, positive affect, and interpersonal relations. The respondents were asked to mark how often they have felt in a certain way during the last month. Sample items include: “I was bothered by things that usually don't bother me,” “People were unfriendly,” and “I had crying spells.” Cronbach's alpha of the depression scale was at an acceptable level (α = 0.85).

### Statistical Analysis

**Figure 1:**
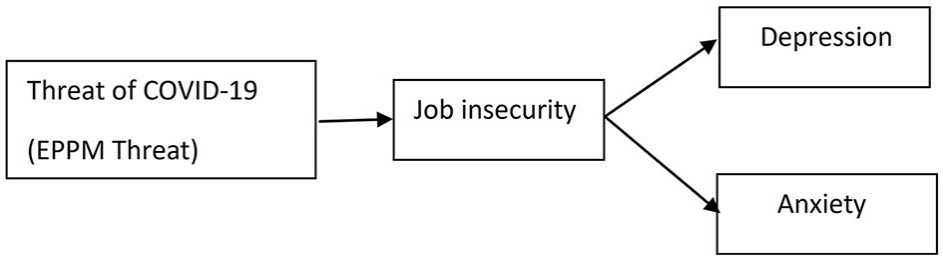
Research model of the study.

The research model was constructed to explain the impact of EPPM threat on anxiety and depression, the impact of job insecurity on anxiety and depression, and their mediating effect of job insecurity (Figure 1). SEM software AMOS version 23 was used to conduct the analysis. Statistical analysis was performed on a sample of 347 respondents after eliminating 66 respondents because of missing values. The measurement tool was validated and tested for reliability. CFA with maximum likelihood estimation was performed for the observed model. CFA is a first step to assess whether the data fit a hypothesized measurement model during a structural equation model analysis. Next, the goodness-of-fit indices χ2/df (normed chi-square statistic); GFI (goodness-of-fit index); RMR (root-mean-square residual); RMSEA (root mean square error of approximation); NFI (normed fit index); TLI (Tucker–Lewis Index), CFI (comparative fit index) were calculated to assess model goodness of fit. Absolute fit indices determine how well the presumed model fits the data. Next, composite reliability (CR), average variance extracted, and correlation matrix were determined, confirming convergent and discriminant validity, with results presented in **Table 2**. Common latent factor (CLF) method captured the common variance among all observed variables in the model. Latent variables are represented as circles, whereas variances are drawn as double-headed arrows into an object. Squares represent tested items, and e is a measurement error in each item. In the structural model testing with SEM, relationships between EPPM threat, job insecurity, and anxiety and depression were examined. Path analysis has been conducted, and standardized parameter estimates, standard errors, and *p*-values for the structural model were computed. They are summarized in **Table 3**.

## Results

The data received were cleaned, missing values excluded, and prepared for further testing. Analysis was performed on the sample of 347 respondents. In the structural model testing with SEM, relationships between EPPM threat, job insecurity, and anxiety and depression were examined. Path analysis has been performed, and standardized parameter estimates, standard errors, and *p*-values for the structural model were computed. The initial confirmatory factor analysis test showed that the model needs further improvements (χ2/df = 3.754; CFI = 0.798; SRMR = 0.08; RMSEA = 0.053). χ2/degrees of freedom ratio is higher than the recommended value of 3 (Hair et al., 2013). SRMR is equal to 0.08, which is the minimum requirement of good model fit (Byrne, 2010). CFI illustrates good model fit with a value below 0.95. While RMSEA indicates the need for model fit improvements with a value higher than the threshold of 0.05, it still meets the suggested level of < 0.08 (Ferdinand, 2002). Factor loadings of below 0.55 were deleted (Fornell and Larcker, 1981), consequently improving the model fit.

Furthermore, because of the correlation found between some components, the covariances were added. Also, the pair of items within depression errors (3–5, 6–9) was correlated. Such manipulation was justified by Byrne (2010) because of synonymous formulation. The covariance of within item errors resulted in the achievement of excellent model fit. That is indicated with SRMR (0.053), which was below 0.08, as suggested by Byrne (2010). Simultaneously, the value of RMSEA was also below 0.08. According to the requirement for χ2/df (Hair et al., 2013), the ratio fits within the required range of 1–3. As for the CFI, the index is above the threshold of 0.9 (0.952), thus achieving an excellent model fit (χ2/df = 2.519; CFI = 0.952; SRMR = 0.053; RMSEA = 0.039) as per Hu and Bentler (1999) and Byrne (2010). Table 1 indicates the model fit parameters.

**Table 1:** Model fit measures.

**Measure**	**Estimate**	**Threshold**	**Interpretation**
CMIN	1088,061	–	–
DF	432	–	–
CMIN/DF	2.519	Between 1 and 3	Excellent
CFI	0.952	>0.95	Excellent
SRMR	0.053	<0.08	Excellent
RMSEA	0.039	<0.06	Excellent

*PClose 1 >0.05 Excellent*.

*χ2/df, normed chi-square statistic; GFI, goodness-of-fit index; RMR, root-mean-square residual; RMSEA, root mean square error of approximation; NFI, normed fit index; TLI, Tucker–Lewis Index; CFI, comparative fit index*.

Convergent and discriminant validity checks of the measurement model were performed. The average variance extracted (AVE) coefficients are higher than the minimum requirement of 0.5. Moreover, AVE of all the variables except for the EPPM threat is above 0.6. CR was also calculated for each construct, with all values above 0.8. Obtained CR coefficients values surpass the threshold of 0.6, indicating consistency. That confirms the convergent validity of all the variables. Discriminant validity was also established by comparison of the AVE and maximum shared variance (MSV).

AVE for each of the constructs is higher than MSV, confirming discriminant validity. Validity results together with the factor correlation matrix with the square root of AVE are displayed in Table 2. Factor correlation values are located diagonally in the table.

Table 3 illustrates the standard regression weights between latent variables and observed ones. Items in the constructed survey represent observed components of the latent variables such as Depression, Anxiety, EPPM threat and Job Security. Thus, Anxiety 1 or Depression 18 represents item 1 of the Anxiety construct and item 18 of the Depression construct. All the standard regression weights represented in Table 3 are above 0.5.

**Table 2:** CR, AVE, and correlation matrix.

**Variable**	**CR**	**AVE**	**MSV**	**MaxR(H)**	**EPPM.Threat**	**Depression**	**Anxiety**	**Job.Insecurity**
EPPM.Threat	0.820	0.547	0.005	0.877	**0.739**			
Depression	0.907	0.620	0.612	0.925	0.050	**0.788**		
Anxiety	0.945	0.740	0.612	0.948	0.068	0.782	**0.860**	
Job.Insecurity	0.884	0.720	0.141	0.909	−0.023	0.376	0.249	**0.848**

*The bold values represent square root of the AVE for each of the variable*.

**Table 3:** Standardized regression weights.

			**Estimate**
Depression.18	< -	Depression	0.865
Depression.6	< -	Depression	0.909
Depression.9	< -	Depression	0.68
Depression.19	< -	Depression	0.721
Depression.5	< -	Depression	0.722
Depression.3	< -	Depression	0.76
Anxiety.3	< -	Anxiety	0.909
Anxiety.2	< -	Anxiety	0.884
Anxiety.1	< -	Anxiety	0.904
Anxiety.4	< -	Anxiety	0.875
Anxiety.6	< -	Anxiety	0.831
Anxiety.7	< -	Anxiety	0,821
EPPM.Threat.4	< -	EPPM.Threat	0,881
EPPM.Threat.6	< -	EPPM.Threat	0,886
EPPM.Threat.5	< -	EPPM.Threat	0,768
Job.Security.1	< -	Job.Security	0,893
Job.Security.4	< -	Job.Security	0,891
Job.Security.3	< -	Job.Security	0,702
EPPM.Threat.1	< -	EPPM.Threat	0,533

CFA analysis was followed by structural equation model testing (SEM). The hypothesized relationships between EPPM threat, Depression, Anxiety, and Job Security were examined for the model fit. The results indicated that the hypothesized model requires improvements. Based on modification index (MI) illustrating the improvements in chi-square (CMIN), there are two options to improve model fit: 1) to add the path between Anxiety and Depression and 2) to add covariance between e2 and e3. The Anxiety and Depression model path would result in model overfit and inability to calculate chi-square and degree of freedom. Moreover, CFI would increase to 1, indicating overfit as all the factors are linked. Thus, to achieve a good model fit, the covariance between e2 and e3 was added. As a result, the MI indicated chi-square (CMIN) improvements of 323.

**Figure 2:**
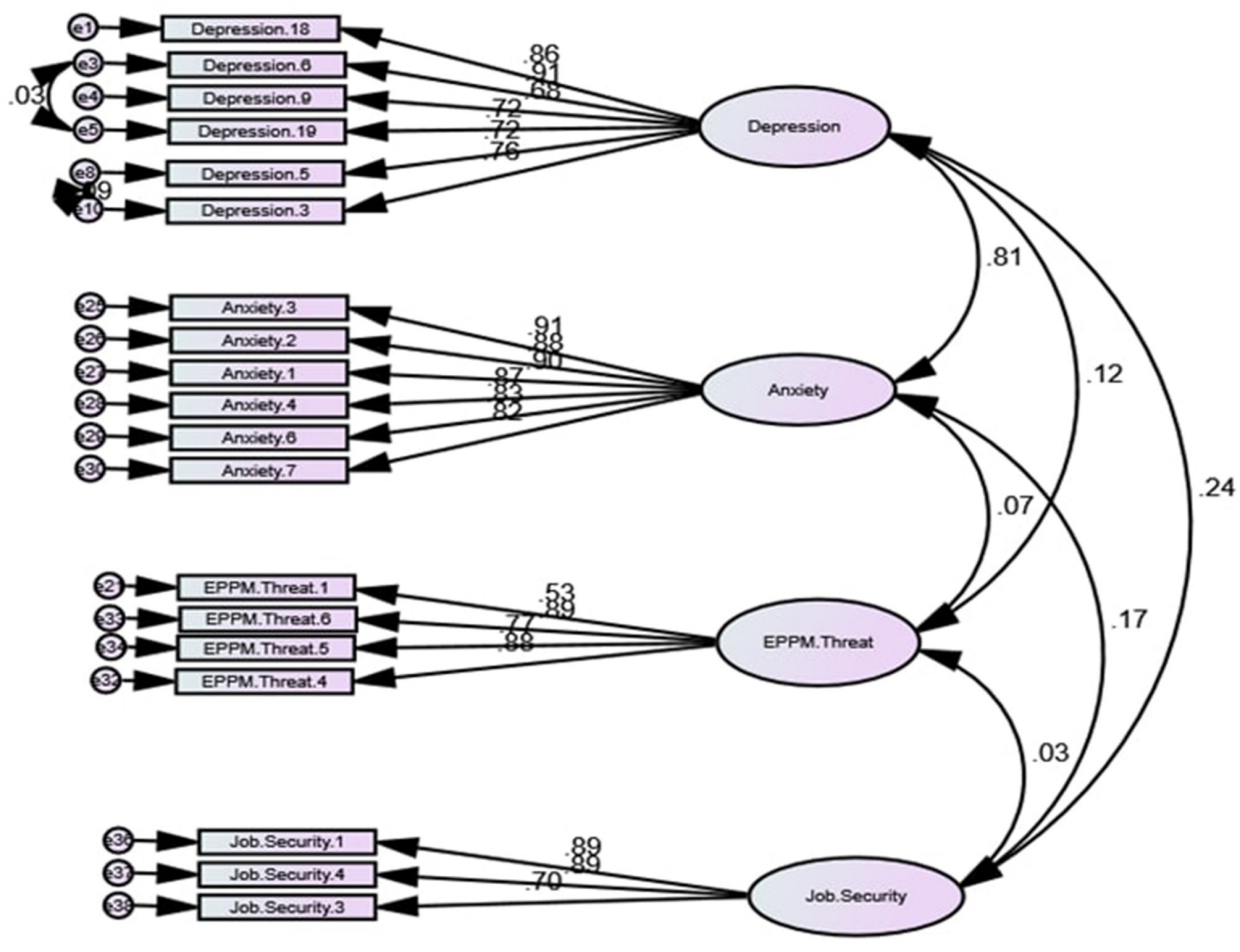
Measurement model.

Before removing the path between EPPM threat and Job Security, an additional check of the indirect impact of Job Security on the relationship between EPPM threat and two other variables, Depression and Anxiety, was performed. The bootstrap significance test (*p* > 0.05) detected no mediation effect, confirming that the exclusion of the path will not impact dependent variables. Thus, the insignificant path between EPPM threat and Job Security has been removed due to the poor connection and insignificant *p*-value significance (*p* = 0.573), resulting in the rejection of Hypothesis 1. The above-mentioned manipulations helped to achieve an excellent model fit (χ2/df = 0.417; CFI = 1; SRMR = 0.012; RMSEA = 0). The improved model is illustrated in Figure 2.

**Figure 3:**
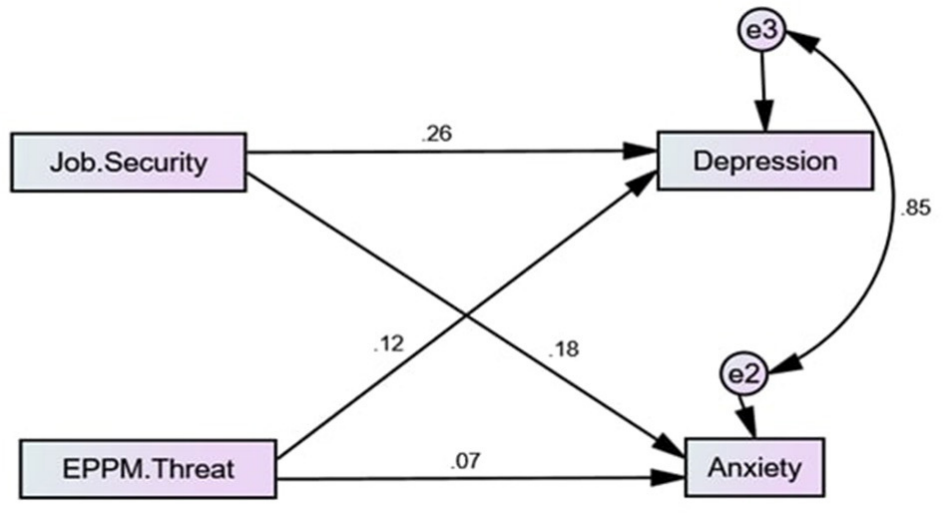
Improved structural model.

As part of path analysis between the variables, SEM testing was performed. According to the results, there is a positive and highly significant impact of Job Security on Depression (β = 0.293; *p* <0.001) and Anxiety (β = 0.213; *p* = 0.001). Thus, both Hypotheses 2 and Hypotheses 3 can be accepted. The SEM model is depicted in Figure 3.

The relationship between EPPM threat and Anxiety stated in Hypotheses 5 was insignificant with *p*-value above the set threshold, resulting in hypothesis rejection. On the other hand, the positive β-coefficient (0.151) with a significant *p*-value of 0.02 confirms the effect of EPPM threat on Depression. Thus, Hypotheses 4 is accepted. The tested and obtained regression results are displayed in Table 4.

**Table 4:** Standardized parameter estimates, standard errors, and *p*-values for the structural model.

			**Estimate**	**S.E**.	**C.R**.	***P***
Depression	< -	Job.Security	0.293	0.059	4.929	^***^
Depression	< -	EPPM.Threat	0.151	0.065	2.324	0.02
Anxiety	< -	EPPM.Threat	0.098	0.072	1.375	0.169
Anxiety	< -	Job.Security	0.213	0.065	3.248	0.001

*** *p-value < 0.001; SRW, standardized regression weights; URW, unstandardized regression weights; CR, critical value*.

## Discussion

In the COVID-19 pandemic economic situation, concerns regarding long-term fiscal outlook are justified, as rise in job insecurity and economic turmoil is spilling over, affecting the mental health of citizens. Such circumstances bring to attention the consequences of maladaptive coping with unemployment on psychological and mental health. We theorized that adverse emotional reactions to the perceived threat account for maladaptive coping, while positive self-efficacy and response efficacy beliefs lead to assuming adaptive damage control actions, affecting the level of depression and underlying anxiety. The reasoning for assuming such approach stressing the role of threat and coping mechanisms stems from the conclusions of previous studies on the relationship of infectious disease to coping behaviors (Rubin, 2009; Bish and Michie, 2010; Teasdale et al., 2012; Van Bavel et al., 2020). During infectious respiratory pandemics, psychological conditions and protective responses help brief on effective risk communication and interventions directing behavioral modifications. Such study is concentrated on risk assessment through normative cognitive processes, such as measuring the perceived plausibility of infection, while affective processes are examined by measuring the intuitive and experiential dimensions of worry and anxiety. Several studies so far explored the relationship between psychological and behavioral indications during preceding epidemics (Liao et al., 2014), and we endeavored to assess associations between distinct cognitive and affective responses to risk reduction measures based on self-reported protective behaviors enriching the existing theoretical and empirical body of knowledge on the subject.

The findings are consistent with cross-national evidence on adverse mental health outcomes generated during the assessment of coronavirus uncertainty repercussions (Godinic et al., 2020; Aguiar-Quintana et al., 2021; Blanuša et al., 2021; Ganson et al., 2021; Ruffolo et al., 2021). Blanuša et al. (2021) found that the distress increases proportionately to the rise of job uncertainty and fear of COVID-19. Nelson and Kaminsky (2020) asserted that the ongoing crisis concerning health and social and monetary security significantly contributes to intense anxiety and depression. Dramatic disruptions in the work setting cause significant adverse economic, social, and psychological fallout, resulting in psychological and physical impairment (Stanisławski, 2019). As the phenomenon is of major concern for organizational psychology, the negative effect of job uncertainty on anxiety and depression levels of an employee has previously been extensively investigated (Bert et al., 2021; Ganson et al., 2021; San Too et al., 2021), and this study corroborates the relationship in the novel context of coronavirus among United States workers. Therefore, Hypotheses 2 and 3 stating that job insecurity leads to depression and anxiety are accepted. Many respondents reported harmful emotional and affective effects of sudden and extreme lifestyle changes introduced by authorities to constrain infectious COVID-19, such as compulsive fear, severe anxiety, and depressive reflection. Such negative appraisals resulted mainly from job uncertainty because of economic slowdown, recession, paycheck reduction, growing unemployment, and poverty. Although enforcing precautionary measures, such as social distancing, mobility restrictions, large gatherings regulation, introducing online schooling and remote working, and implementing educational protective campaigns, for most states, resulted in temporary suppression of the pandemic, and a pervasive pattern of maladaptive coping strategies among the survey participants was recorded. Expectedly, the respondents showed a high degree of threat awareness and concern over recommended defense response effectiveness. However, even those fully compliant with the instructions were not excluded from experiencing an existential crisis, exhaustion, inability to concentrate, isolation, worry, and depression.

Following EPPM, we supposed that individuals perceiving themselves to be highly exposed to the virus and aware of the severity of the threat, with the high-efficiency appraisal of defensive response usefulness, would develop adaptive coping strategies directing positive behavior change. However, the results of this study show that EPPM does not impact job insecurity. For the second hypothesis, we assumed job security would impact depression, as uncertainty and intensive fear leave a strain on psychological well-being. Stressful and trauma-inducing events, such as significant catastrophes or natural disasters, economic breakdowns or global health threats, marked by intolerable uneasiness and confusion, predispose individuals to anxiety disorders (Maslow, 1962, 1971; Boya et al., 2008; Burgard et al., 2012; Caramanica et al., 2014; IMF, 2020; Li et al., 2020). In this, the results corroborate the findings of Leininger and Kalil (2014) and Gladstone et al. (2005). Job insecurity, in particular, comprises cognitive and affective dimensions. Cognitive refers to the perception of the prospect negative changes by employees, and affective encompasses the experiential state of anxiousness over possible job loss (Huang et al., 2012), whereby the presence of each leads to severe psychological damage (Bünnings et al., 2017). Therefore, the hypothesis is accepted. Furthermore, in line with the previous results generated by studies of Godinic et al. (2020), LaMontagne et al. (2020), and Rossi et al. (2020), the results confirm the detrimental effect of job security on anxiety. Additionally, the findings suggest that the EPPM threat has a positive impact on depression. Therefore, health campaigns educating the general public on prevention, psychological support, and therapeutic advice *via* online channels would transform to a positively adoptive response combating depression.

At the same time, psychologically weaker and vulnerable personalities already predisposed to mood disorders would be negatively afflicted by novel measures, thus developing a negative fear-related response in the form of adverse coping behaviors. Finally, the EPPM threat has a positive impact on anxiety. Often, media and society induce fear by exposing individuals to provocative photos and information, providing at the same time simple and effective solutions. Depending on the fear-induced degree, one will respond negatively by excessive avoidance, leading to anxious paralysis. On the other hand, in psychologically stronger personalities, danger control adaptive response may lead to decreasing anxiety levels by perceiving response as effective and plausible. Therefore, the hypothesis stating EPPM impacts anxiety was accepted, in line with previous evidence from Lee (2020).

In light of the most recent research on the ongoing hazard, by examining 16 most relevant studies, Pollock et al. (2020) found that insight, information as well as beliefs concerning the threat can act both as facilitators and barriers to administering mental health interventions. Such appraisal is in line with the tenets of EPPM, and the results further support this conjecture. When employees perceive disaster severity and the potency of constraint as both high and effective, the intervention to prevent workplace stressors will have a higher success rate (Stanisławski, 2019). Excessiveness of misinformation from both official and private sources causes doubt and hinders intervention implementation. It is only logical that the exaggerated fear and negative beliefs regarding possible hazard curtailing will cause depression. Depression is characterized by the prevalence of negative emotional copings, such as instability in the sense of self, low mood, inadequate motivation, loneliness, and fearfulness (Stanisławski, 2019), and all of these are perpetuated by pandemic-related uncertainty, especially concerning prolonged recovery and in the absence of disaster precedents with positive aftereffects. The lack of knowledge, inability to render reliable forecasts, and the shortfall of coherent and accurate information on COVID-19 are especially disheartening. Thus, the hypothesis stating that the EPPM threat of COVID-19 leads to depression is accepted, consistent with the findings of Aguiar-Quintana et al. (2021) and Ganson et al. (2021).

Furthermore, the extensive analysis of Pollock et al. (2020) provides an insight into underlying intervention impediments. Workers and organizations are possibly not fully apprehensive of resources required to improve mental resilience, or they cannot foster a positive atmosphere by setting up a psychosocial support system beforehand, such as counseling and psychological aid. Organizations that failed in their preparedness attempts and lack control are likely to have higher stress levels and low response success rates. The lack of adequate infrastructure for carrying out large-scale mental health interventions and shortage of qualified personnel can also be recorded at a state level. When this happens, the organizational issue translates to a full-blown national challenge. However, despite the initial assumption, we did not find the same positive association between the EPPM threat of COVID-19 and anxiety. Only some of the employees reported anxiousness and substantial worry over imminent threat to their social, working, and psychological status and enduring threat to job security.

EPPM posits that the increased threat will elucidate greater fear. The lack of fear appeal may be due to disbelief in the accuracy of risk communication and the misperceived threat severity and susceptibility or overconfident efficacy belief. The anxiety component of EPPM is not raised when people believe that information on emergent and disastrous events, such as the COVID outbreak, are fabricated, amplified, or exaggerated by the government and media outlets to cause chaos and panic, and impose restriction obedience. Furthermore, when employee perception of threat severity is diminished, their anxiety levels are bound to be lesser. Zhao and Wu (2021) found that in such cases, employees rather rely on their social networks for intel, and clues they get may be biased, censored, incorrect, or filtered. Accordingly, in line with the study of Zhao and Wu (2021) on the mediation effect of EPPM, we, too, have failed to establish a meaningful mediation effect of job insecurity between COVID-19 risk perception and anxiety and depression of employees. At the onset of an emergency, marked by a high level of uncertainty, fear may not be the prevailing emotion, as alternative instances of worry, sadness, stress, and depression cannot be precluded and are potentially more significant (Kleinberg et al., 2020; Zhang et al., 2020).

The results are consistent with most of the assumptions discussed in Carmassi et al.'s (2020) analysis of studies on psychological distress risk factors during three Coronavirus outbreaks. Authors who have examined the adverse effects of previous outbreaks shed light on the protective role of psychological support in fostering resilience and reported on the relevance of clear precautionary measures communication during adversity. In line with EPPM, precautionary measures are perceived as relevant, unambiguous, attainable, and effective. Each reduction in these conditions, almost without exception, results in increased anxiety and depression and can, through accumulative and prolonged exposure, further progress in PTSD. Although some studies (Huang et al., 2020; Lai et al., 2020) have reported that the outbreak-related work burden resulted in depression and anxiety, we could not confirm with substantial significance the relationship between the EPPM COVID-19 threat and anxiety. EPPM advocacy should draw from the cognizance of the risk factors interacting with increased stress and concern, such as lack of support and resources, to better manage the crisis and advance prevention (Zhou, 2021). In terms of disaster preparedness, anxious employees are often less likely to prepare, as they are less susceptible to risk and are prone to avoidance and emotional coping (Mishra and Suar, 2012; McNeill et al., 2016). When experiencing extreme anxiousness or depression, employees tend to shut down and disregard instructions, as they lack the incentive to act defensively, and each action may seem meaningless. Healthcare adversaries should be attentive and use appropriate wording when managing fear-driving campaigns. They should be careful not to induce negative emotions of distress and fright when raising awareness, stimulating protective motivation and healthy coping, and retaining subtle balance while communicating threat severity and safety recommendations.

## Conclusion

We find the multidisciplinary psychological, psychiatric, and phenomenological approach to essential dimensions of human experience and illuminating discussion of basic features of mental disorders to be rather informative and best suited in light of the COVID-19 situation. This study contributes to the apprehension of the repercussions of major environmental disruptions on normal human functioning, and it investigates the effects of self-reported protective behaviors to risk perception. Even though the study is valuable, several study limitations exist. First, self-reported measures were employed to assess the variables of the model. Future studies could use observations and reports of mental health professionals on the mental states of the studied sample. Next, the study was cross-sectional, with data collected at one point in time. Future studies can be longitudinal to evaluate how anxiety and depression change with decreased fear and insecurity, and if the relationships between the variables are significant in the COVID-19 pandemic aftermath. Another limitation pertains to the generalizability and applicability of the results. Because of high contagion and data availability on psychological COVID-19-precipitated impairment, the authors only focused their efforts on United States workers. Thus, there is a question of whether the study findings are generalizable. We assume that the findings are applicable in other states and contexts, given that the results concerning the relationship between EPPM threat and job uncertainty, depression, and anxiety are concerned with the more general nature of causality between fear, emotional responses, and coping strategies.

Nevertheless, the study research model should be validated in other contexts and on other samples. This study considers the EPPM threat an additional critical risk factor influencing the psychological well-being of workers and, thus, falls under the comprehensive scientific and clinical action-oriented undertaking to explain and understand the versatile nature of the ongoing disaster. The study contributes to understanding the underlying mechanisms of coping behavior as possible antecedents to mental disorders. When subjected to stressful events, heightened psychological arousal causes physical and psychological challenges of affected employees to manifest as behavioral issues. EPPM threat stems from the presumption that employees under stressful conditions engage in emotional regulation and stress management by forming opposing cognitive and affective problem-solving coping strategies. Depending on the appraisal of the upcoming threat and EPPM campaign message interpretation, workers rise to the occasion by engaging in danger control on the spectrum ranging from effective preservation to adjustment to trauma. The research study contributes to the existing theories on the relationship between threats to financial and psychological welfare and psychological impairment, which are often consulted when creating intervention policies. Moreover, the negative account on the association between EPPM threat and anxiety can be of great value for the assessment of the EPPM model validity and applicability in the context of health disasters, granting many academics regard the two constructs as positively associated and consequential (Hayes-Skelton and Graham, 2013; Panah et al., 2018; Lee, 2020). As such, the study can be integrated with the emerging academic literature on COVID-19.

Moreover, the model is pragmatic in that it can be practically employed during the design and execution stages of large-scale psychological policy interventions aimed at supporting mental health resilience in most work settings. Model is meant to be used for converging the occupational with epidemiologic communication regarding the current and imminent mental healthcare concerns. Persuasive messages concerning the appropriate and effective methodology to foster the psychological resilience of employees can, based on the findings, be used to induce protective motivation and appropriate damage-averting response. EPPM campaigns are employed to inform about the dangers of long-term exposure to workplace stress and contagion risks and on the threat COVID-19 poses to job certainty. Furthermore, the explanation of the authors on how external stimuli shape individual internal states can help steer organizations and managers toward ethical decision-making and healthy coping strategies. It is not limited to a particular national, professional, or work setting.

## Data Availability Statement

The raw data supporting the conclusions of this article will be made available by the authors, without undue reservation.

## Ethics Statement

The studies involving human participants were reviewed and approved by Jiangsu University. Written informed consent for participation was not required for this study in accordance with the national legislation and the institutional requirements.

## Author Contributions

BO conceived the idea, contributed to the design of the study, involved in all steps of the research process, wrote the first setup, and draft of the study. JD contributed to the study design, data acquisition, result interpretation, and drafted the manuscript. DG made a substantial, direct and intellectual contribution to the study and participated in writing, and editing the manuscript. DT researched the statistical methods and contributed to analysis and result interpretation. MB contributed to the design of the study, manuscript editing, and data collection. All authors approved the manuscript and agreed to be accountable for all aspects of the study.

## Conflict of Interest

The authors declare that the research was conducted in the absence of any commercial or financial relationships that could be construed as a potential conflict of interest.

## Publisher's Note

All claims expressed in this article are solely those of the authors and do not necessarily represent those of their affiliated organizations, or those of the publisher, the editors and the reviewers. Any product that may be evaluated in this article, or claim that may be made by its manufacturer, is not guaranteed or endorsed by the publisher.
