# Supplementary material for: The Threat of COVID-19 and Job Insecurity Impact on Depression and Anxiety: An Empirical Study in the USA
Source: Front Psychol. 2021 Aug 13;12:648572. doi: 10.3389/fpsyg.2021.648572 (PMC8411708; doi:10.3389/fpsyg.2021.648572)
Supplement: Supplementary file 1 [file Data_Sheet_1.docx]

Scales used in the questionnaire

1. EPPM threat: Please indicate the degree to which you agree or disagree with each of the following statements

Strongly disagree Disagree

Neither agree or

disagree Agree Strongly agree


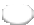

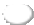

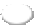

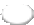

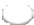


I am at risk for COVID- 19.


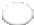

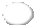

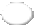

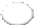

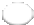
It is likely that I will develop COVID-19.


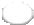

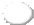

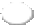

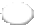

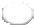


It is possible that I will develop COVID-19.

I believe that COVID-19

is a severe health
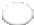

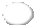

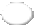

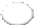

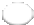
 problem.


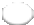

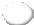

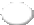

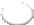

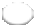


I believe that COVID-19 is a serious threat to my health.

I believe that COVID-19 is a significant disease.

1.
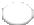

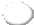

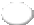

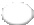

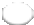
Anxiery: Over the last month, how often have you been bothered by the following problems?


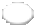

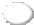

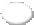

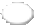

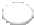
Not at all Several days Half of the days Over half the days Nearly every day


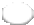

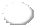

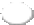

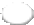

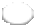


Feeling nervous, anxious, or on edge

Not being able to stop or control worrying


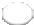

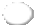

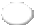

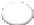

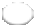


Worrying too much about different things


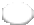

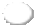

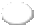

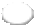

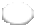
Trouble relaxing
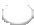

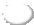

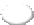

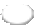

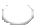


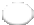

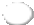

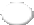

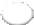

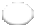


Being so restless that it's hard to sit still

Becoming easily annoyed or irritable


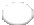

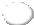

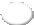

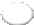

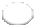


Feeling afraid as if something awful might happen

1. Job insecurity: Please indicate the degree to which you agree or disagree with the following statements in the context of the last month

Strongly disagree Disagree

Neither agree or

disagree Agree Strongly agree


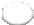

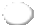

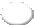

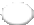

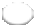


Chances are, I will soon lose my job.


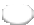

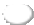

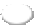

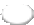

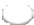
I am sure I can keep my job.


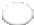

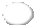

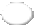

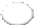

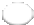


I feel insecure about the future of my job.

I think I might lose my job in the near future.

1.
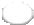

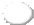

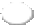

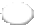

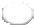
Depression: Please mark how often you have felt this way during the last month.

Occasionally or a

Rarely or none of the time (less than a week)

Some or a little of the time (less than half of the month)

moderate amount of time (half of the month)

Often (more than half of the month)

Most or all of the time (the whole month)


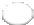

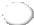

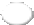

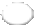

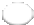


I was bothered by things that usually don’t bother me.


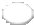

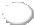

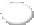

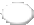

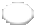
I did not feel like eating; my appetite was poor.


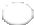

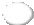

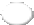

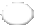

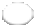


I felt that I could not shake off the blues even with help from my family or friends.

I felt I was just as good as other people.

I had trouble keeping my mind on what I was doing.

I felt depressed.

I felt that everything I did was an effort.

I felt hopeful about the future.

I thought my life had been a failure.

I felt fearful.

My sleep was restless.

I was happy.

I talked less than usual.

I felt lonely.

People were unfriendly.

I enjoyed life.

I had crying spells.

I felt sad.

I felt that people dislike me.

I could not get “going.”
